# Towards The Automated, Empirical Filtering of Drug-Drug Interaction Alerts in Clinical Decision Support Systems: Historical Cohort Study of Vitamin K Antagonists

**DOI:** 10.2196/20862

**Published:** 2021-01-20

**Authors:** Emmanuel Chazard, Augustin Boudry, Patrick Emanuel Beeler, Olivia Dalleur, Hervé Hubert, Eric Tréhou, Jean-Baptiste Beuscart, David Westfall Bates

**Affiliations:** 1 Univ. Lille, CHU Lille, ULR 2694 - METRICS, CERIM, Public health dept, F-59000 Lille France; 2 Division of Occupational and Environmental Medicine Epidemiology, Biostatistics and Prevention Institute University Hospital Zurich & University of Zurich Zurich Switzerland; 3 Division of General Internal Medicine Department of Medicine Brigham and Women's Hospital, Harvard Medical School Boston, MA United States; 4 Clinical Pharmacy Research Group Louvain Drug Research Institute Université catholique de Louvain Brussels Belgium; 5 Pharmacy department Cliniques universitaires Saint-Luc Université catholique de Louvain Brussels Belgium; 6 Univ. Lille, CHU Lille, ULR 2694 - METRICS, F-59000 Lille France; 7 Department of Medical Information Centre Hospitalier de Denain Denain France

**Keywords:** decision support systems, clinical, clinical decision support system, medical order entry system, computerized physician order entry, over-alerting, alert fatigue, drug-drug interaction, drug-related side effects and adverse reactions, vitamin K antagonist, anticoagulants

## Abstract

**Background:**

Drug-drug interactions (DDIs) involving vitamin K antagonists (VKAs) constitute an important cause of in-hospital morbidity and mortality. However, the list of potential DDIs is long; the implementation of all these interactions in a clinical decision support system (CDSS) results in over-alerting and alert fatigue, limiting the benefits provided by the CDSS.

**Objective:**

To estimate the probability of occurrence of international normalized ratio (INR) changes for each DDI rule, via the reuse of electronic health records.

**Methods:**

An 8-year, exhaustive, population-based, historical cohort study including a French community hospital, a group of Danish community hospitals, and a Bulgarian hospital. The study database included 156,893 stays. After filtering against two criteria (at least one VKA administration and at least one INR laboratory result), the final analysis covered 4047 stays. Exposure to any of the 145 drugs known to interact with VKA was tracked and analyzed if at least 3 patients were concerned. The main outcomes are VKA potentiation (defined as an INR≥5) and VKA inhibition (defined as an INR≤1.5). Groups were compared using the Fisher exact test and logistic regression, and the results were expressed as an odds ratio (95% confidence limits).

**Results:**

The drugs known to interact with VKAs either did not have a statistically significant association regarding the outcome (47 drug administrations and 14 discontinuations) or were associated with significant reduction in risk of its occurrence (odds ratio<1 for 18 administrations and 21 discontinuations).

**Conclusions:**

The probabilities of outcomes obtained were not those expected on the basis of our current body of pharmacological knowledge. The results do not cast doubt on our current pharmacological knowledge per se but do challenge the commonly accepted idea whereby this knowledge alone should be used to define when a DDI alert should be displayed. Real-life probabilities should also be considered during the filtration of DDI alerts by CDSSs, as proposed in SPC-CDSS (statistically prioritized and contextualized CDSS). However, these probabilities may differ from one hospital to another and so should probably be calculated locally.

## Introduction

Vitamin K antagonists (VKAs) in general and warfarin in particular are among the most frequently prescribed anticoagulants worldwide [1]. These drugs are used in the primary or secondary prevention of all types of thrombosis [1-3]. However, VKAs are associated with a significant risk of adverse events, due to their narrow therapeutic window, inter- and intra-individual variability, and numerous drug-drug interactions (DDIs) [1,4,5]. The international normalized ratio (INR) is an index of an anticoagulant’s effectiveness and the risk of adverse events. In most indications, the INR should be between 2 and 3 [4,6]. Frequent, close monitoring of the INR is therefore essential, especially if the patient undergoes a change in drug treatment or lifestyle (diet, alcohol intake, etc) or develops new comorbidities [5,7,8].

As the list of drugs that interact with warfarin continues to grow [5], clinicians must be vigilant when initiating treatment with a VKA or when modifying drug prescriptions in VKA-treated patients [1,5]. Although VKAs are not the only anticoagulants concerned with the broader problem of DDI prevention [1], we focused on the members of this drug class because their biological activity can be easily measured.

Clinical decision support systems (CDSSs) provide valuable assistance with VKA prescription because of the large number of potential DDIs [9]. In the setting of computerized physician order entry, the CDSS will indicate potential DDIs (especially for new drug prescriptions) via pop-up alerts. In turn, the alerts are based on DDI rules, which typically involve a pair of interacting drugs and a potential outcome. Whenever the two drugs are present, the DDI alert pops up and highlights the potential outcome [10].

If the number of DDIs is large, however, the resulting over-alerting [11-15] may produce “alert fatigue” [11], a mental state close to overwork caused by the clinician's exposure to a continuous flow of alerts, regardless of whether or not they are relevant [11-16]. On average, only 5%-10% of these alerts are taken into account by the clinician and prompt him or her to reassess the drug prescription [17,18]. Alert fatigue can contribute to physician burnout and has important safety implications because it can cause physicians to ignore even the most important warnings.

Several approaches to decreasing over-alerting and alert fatigue have been developed and tested. These include (1) changing the way alerts are displayed [19-25], (2) refining the alerts’ relevance by filtering them according to clinical veracity [10,11,17,20,21,26-29] or postalert quality assessment by a group of practitioners [29], and (3) managing chronological aspects [19-21,23,24,30]. It has also been suggested that the relevance of alerts can be increased by taking into account the level of evidence for the DDI [20,21] and the seriousness of the outcome [10,17,20,21,27,29,31]. Although this approach appears to improve the situation [10,29,31], experts continue to disagree about how the DDI rules should be classified and how alerts should be displayed [10,32,33].

Another approach involves calculating the likelihood of a given outcome when the DDI rule’s criteria are met; the rules could be turned off if the likelihood is low. This feature has been requested by physicians [20,21,27] and has been theoretically specified as a “statistically prioritized and contextualized CDSS” (SPC-CDSS) [34]. In these CDSSs, the conditional empirical probabilities of adverse drug events (ADEs) are computed by reuse of electronic health records (EHRs) [35,36].

The strategic objective of this study was to generate empirical evidence in favor of SPC-CDSSs. The operational objective was to compute empirical conditional probabilities of outcome for VKA-related DDI prevention rules, via data reuse of EHRs.

## Methods

### Overview

This was a retrospective cohort study. The study population comprised all the inpatient stays from 2007 to 2014 in a set of French, Danish, and Bulgarian hospitals (see Inpatient Stays section) participating in the European “Patient Safety through Intelligent Procedures“ (PSIP) project [37]. A set of DDI rules was defined, including causes (a VKA and another drug) and potential outcomes (VKA potentiation or inhibition, as defined in the Set of DDI Rules section). The causes and the potential outcomes were retrospectively tracked over time in the data set, and the probability of each outcome was estimated automatically for each DDI rule.

### Inpatient Stays

We reanalyzed 96,378 inpatient stays in a French community hospital, 53,635 inpatient stays in a group of Danish community hospitals, and 6880 inpatient stays in a Bulgarian hospital. Only stays with at least one laboratory INR result and at least one day with VKA administration were included. Those data had been collected exhaustively during routine patient care. The available data [9] included (1) demographic and administrative information (eg, age, gender, and dates), (2) diagnoses coded according to the International Statistical Classification of Diseases and Related Health Problems, 10th Revision [38], (3) daily drug administrations, encoded using the Anatomical Therapeutic Chemical (ATC) Classification System terminology [39], and (4) laboratory results encoded using the Clinical Nomenclature for Properties and Units terminology [40].

### Set of DDI Rules

We used the combined results of three literature reviews (Holbrook et al [7], Nutescu et al [5], and Di Minno et al [1]) to identify DDI rules involving VKAs. After deduplication, a list of 149 DDIs (available in Multimedia Appendix 1) was created. We then mapped the drug names to ATC codes [39] by taking into account the active substances and the administration route. The ATC mapping was inclusive and, when appropriate, also involved ATC codes relating to drug combinations.

Of the 149 DDIs, 7 were excluded because they corresponded to drugs without ATC terms. Two drugs had the same ATC code (amoxicillin + tranexamic acid, and amoxicillin + clavulanate) and were therefore combined in 1 DDI. The remaining drugs were variously analgesics, antipyretics, and immunological agents (n=21), anti-infectives (n=47), cardiovascular and anti-hypertensive drugs (n=29), central nervous system drugs (n=19), and other drugs (n=25). Ultimately, we obtained 107 drugs that might potentiate VKAs and 34 drugs that might inhibit VKAs (including 4 drugs that belonged to both categories). A final set of 141 DDI rules was obtained for drug administration. The same number of rules was obtained for drug discontinuation, leading to 2×141 rules in total.

We then obtained DDIs, in the form “VKA & administration of DrugX → outcome” and “VKA & discontinuation of DrugX → reverse outcome,” where the “DrugX” term was a drug that potentially interacted with VKAs, and the “outcome” term was defined as VKA potentiation (INR≥5) or inhibition (INR≤1.5).

### Statistical Analysis

In descriptive analyses, qualitative variables were reported as the number and percentage for each category, and quantitative variables were reported as the mean and standard deviation (SD) for symmetric data distributions or the median and interquartile range (IQR) for asymmetric data distributions.

**Figure 1:**
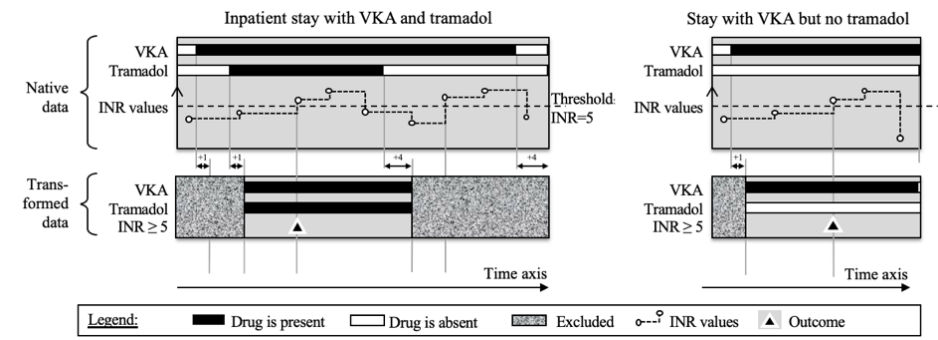
Data management: definitions of the inpatient stays included in the analysis. Time advances from left to right. INR: international normalized ratio. VKA: vitamin K antagonist.

The main objectives of the statistical analysis were to follow up each inpatient stay in which a VKA was administered, detect outcomes over time, and estimate odds ratios (ORs) for the second drug in the DDI rule. The following procedure was applied for each DDI rule. The “VKA & tramadol → INR≥5” rule serves here as an example. Figure 1 shows the data transformation process for a hospital stay with VKA and tramadol (an “exposed stay,” left side) and a stay with VKA but no tramadol (a “nonexposed stay,” right side). The observation periods were designed to reflect each drug’s onset of action and postdiscontinuation duration of action. An “exposed” inpatient started the day after the two drugs had been administered together and ended 4 days after the first of the two was discontinued or after both were discontinued on the same day. A “nonexposed” inpatient started on the day after the VKA had been administered and stopped 4 days after the VKA had been discontinued. The observation period was searched for the outcome (Figure 1).

For each drug, we used the same approach to test whether drug discontinuation would lead to the opposite outcome. For instance, the “VKA & tramadol → INR≥5” rule also enabled us to test the “VKA & tramadol discontinuation → INR≤1.5” rule.

We first computed the unadjusted OR (95% confidence limits [CLs]) for the exposure and the outcome, using the Fisher exact test [41]. We then performed a multivariable logistic regression to predict the outcome. The covariates were the studied drug, age, albuminemia, pre-albuminemia, creatininemia, aspartate transaminase/alanine transaminase (ASAT/ALAT) levels, thyroid stimulating hormone (TSH) level, and N-terminal-pro brain natriuretic peptide (Nt-proBNP) (the last five of these covariates are surrogate markers for malnutrition, kidney failure, liver failure, dysthyroidism, and heart failure, respectively). We thus obtained the adjusted OR (95% CLs). Lastly, the model’s covariates were selected in a stepwise procedure, yielding the “stepwise OR” (95% CLs) [42].

Quantitative variables were placed in classes when the effect was not linear (“ref” denotes the reference class): Age was classified as “<70” (ref), “70-79,” and “≥80”. The albuminemia was classified in g/L as “<30” and “≥30” (ref). Pre-albuminemia was classified in g/L as “<0.07,” “0.07-0.10,” and “≥0.11” (ref). Creatininemia was classified in mg/L as “≤15” (ref), “16-24,” and “≥25”. ASAT/ALAT levels were classified in IU/L as “<250” (ref) and “≥250”. TSH levels were classified in mU/L as “0.5-5” (ref) and “<0.5 or >5”. Lastly, Nt-proBNP was classified in pg/mL as “<450” (ref) and “≥450”. We inferred missing values with normal (reference) values. All statistical analyses were performed with R software (R Foundation for Statistical Computing).

### Ethics

In line with the French, Danish, and Bulgarian legislations on reuse of deidentified data collected during routine medical care, approval by one or more institutional review boards was not required. The study procedures complied with principles outlined in the Declaration of Helsinki.

## Results

### Inpatient Stays

**Figure 2:**
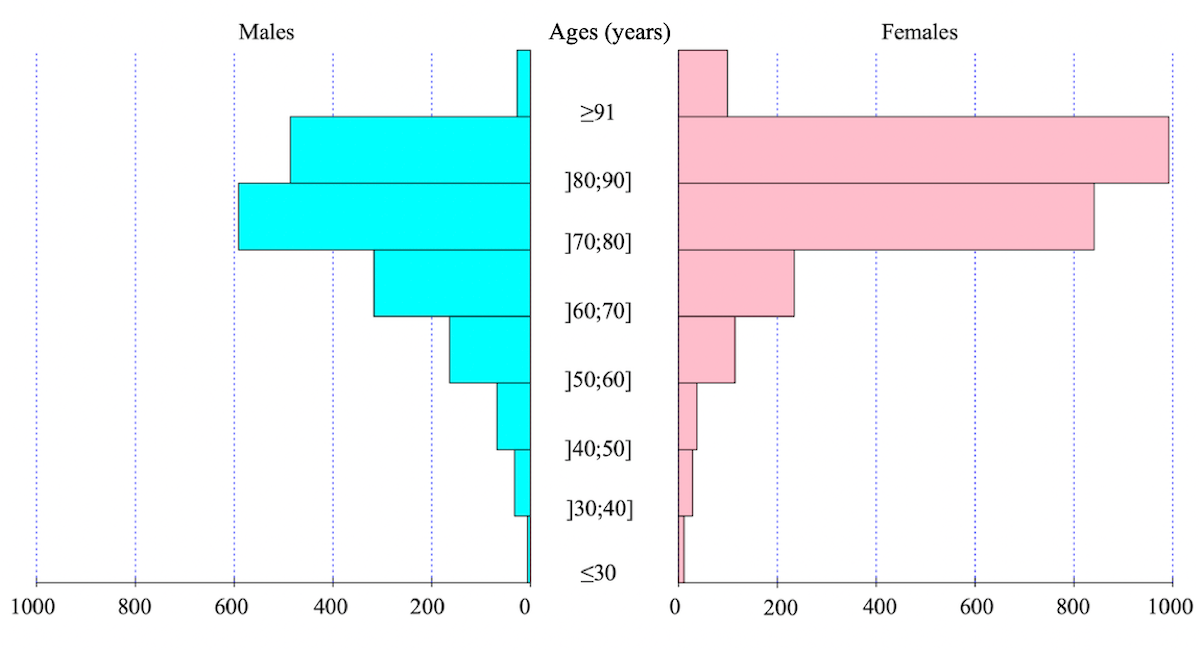
Age pyramid of the patients.

The overall study database included 156,893 inpatient stays, of which the 4047 (2.58%) with VKA administration were analyzed. The mean age (Figure 2) was 75.9 years (SD 12.0), and there were 2356 women (58.2%).

The median length of stay was 9 days (IQR 6-15), and there were 162 in-hospital deaths (4.00%). The VKA administered was fluindione in 3256 cases (80.5%), warfarin in 553 cases (13.7%), acenocoumarol in 227 cases (5.6%), and another VKA or several different VKAs in 11 cases (0.3%).

### Empirical Probabilities of Outcomes for Each DDI Rule

For some DDI rules, fewer than 3 cases of concomitant administration with a VKA were observed in the database, so we did not compute the ORs. The corresponding drugs were as follows:

There were 76 drugs analyzed upon initiation:

- Analgesics, anti-inflammatories, and immunologic agents: cyclosporine, etodolac, interferon, leflunomide, mercaptopurine, nabumetone, phenylbutazone, piroxicam, rofecoxib, sulindac, tolmetin, and trastuzumab.
- Anti-infectives: azithromycin, cefamandole, cefazolin, chloramphenicol, efavirenz, etravirine, fosamprenavir, gatifloxacin, griseofulvin, itraconazole, levamisole, miconazole (vaginal suppositories), nafcillin, nalidixic acid, ribavirin, saquinavir, sulfisoxazole, voriconazole, terbinafine, nevirapine, and ritonavir (the last 3 drugs were involved in 6 DDI rules).
- Cardiovascular drugs: cholestyramine, clofibrate, gemfibrozil, indomethacin, lovastatin, metolazone, ticlopidine, and ubidecarenone.
- Central nervous system (CNS) drugs: chlordiazepoxide, chloral hydrate, disulfiram, entacapone, felbamate, fluvoxamine, methylphenidate, phenytoin, propofol, and trazodone.
- Other drugs: anabolic steroids, cimetidine, danazol, ethanol, etretinate, fluorouracil, gemcitabine, glucagon, ifosphamide, influenzae vaccine, levonorgestrel, paclitaxel, raloxifene, sulfamethoxazole, sulfinpyrazone, tolterodine, topical salicylates, troglitazone, and zafirlukast (sulfinpyrazone was involved in 2 DDI rules).

There were 106 drugs analyzed upon discontinuation:

- Analgesics, anti-inflammatories, and immunologic agents: azathioprine, celecoxib, cyclosporine, etodolac, interferon, leflunomide, mercaptopurine, mesalazine, nabumetone, phenylbutazone, piroxicam, rofecoxib, sulfasalazine, sulindac, tolmetin, and trastuzumab.
- Anti-infectives: azithromycin, cefamandole, cefazolin, chloramphenicol, doxycycline, efavirenz, erythromycin, etravirine, fosamprenavir, gatifloxacin, griseofulvin, isoniazid, itraconazole, levamisole, miconazole (oral gel), miconazole (topical gel), miconazole (vaginal suppositories), moxifloxacin, nafcillin, nalidixic acid, nevirapine, norfloxacin, ribavirin, ritonavir, saquinavir, sulfisoxazole, terbinafine, tetracycline, and voriconazole (nevirapine, ribavirin, and ritonavir were involved in 6 DDI rules).
- Cardiovascular drugs: bezafibrate, bosentan, chelation therapy, cholestyramine, clofibrate, disopyramide, dronedarone, ezetimibe, fenofibrate, fluvastatin, gemfibrozil, indomethacin, lovastatin, metolazone, orlistat, propafenone, quinidine, telmisartan, ticlopidine, and ubidecarenone.
- CNS drugs: barbiturates, carbamazepine, chlordiazepoxide, chloral hydrate, disulfiram, duloxetine, entacapone, felbamate, fluvoxamine, methylphenidate, phenytoin, propofol, quetiapine, ropinirole, sertraline, and trazodone.
- Other drugs: anabolic steroids, cimetidine, danazol ethanol, etretinate, fluorouracil, gemcitabine, glucagon, ifosphamide, influenzae vaccine, levonorgestrel, paclitaxel, raloxifene, sulfamethoxazole, sulfinpyrazone, tamoxifen, tolterodine, topical salicylates, troglitazone, zafirlukast, and oxolamine (sulfinpyrazone was involved in 2 DDI rules).

For other drugs, at least 3 cases of concomitant administration with a VKA were observed.

Upon initiation, 47 drugs did not appear to have a statistically significant impact on the INR:

- Analgesics, anti-inflammatories, and immunologic agents: celecoxib, dextropropoxyphene, methylprednisolone, mesalazine, and sulfasalazine.
- Anti-infectives: amoxicillin, amoxicillin+β-lactamase inhibitor, clarithromycin, ciprofloxacin, dicloxacillin, doxycycline, erythromycin, fluconazole, isoniazid, levofloxacin, miconazole (oral gel), miconazole (topical gel), moxifloxacin, nafcillin, nevirapine, norfloxacin, ofloxacin, ribavirin, ritonavir, terbinafine, tetracycline, tranexamic acid, and trimethoprim;sulfamethoxazole.
- Cardiovascular drugs: bezafibrate, chelators, diltiazem, disopyramide, dronedarone, ezetimibe, fenofibrate, fluvastatin, propafenone, propranolol, quinidine, and telmisartan.
- CNS drugs: barbiturates, carbamazepine, citalopram, duloxetine, fluoxetine, quetiapine, ropinirole, and sertraline.
- Other drugs: acarbose, ketoconazole, sucralfate, and tamoxifen.

Upon discontinuation, 14 drugs did not appear to have a statistically significant impact on the INR:

- Anti-infectives: cloxacillin, dicloxacillin, rifampicin, teicoplanin, tranexamic acid, and trimethoprim;sulfamethoxazole.
- Cardiovascular drugs: candesartan, propranolol, rosuvastatin, and simvastatin.
- CNS drugs: citalopram and fluoxetine.
- Other drugs: acarbose and sucralfate.

The results of the DDI rules for which at least one OR was significant are summarized in Table 1 (for drug initiation) and Table 2 (for drug discontinuation). The “n” column always refers to the number of stays with a VKA and the given drug, although the OR was always estimated for 4047 stays. All of the drugs evaluated in the tables were associated with a protective effect.

**Table 1:** Drugs interacting with VKAs upon initiation and that had at least one significant OR (in all cases, 4047 stays are analyzed).

Drug		Outcome	n	OR^a^ (95% CLs^b^)	Adjusted OR (95% CLs)	Stepwise OR (95% CLs)
**Analgesics, anti-inflammatories, and immunologic agents**						
	Acetaminophen	INR^c^≥5	1023	0.69 (0.56, 0.85)	0.66 (0.53, 0.8)	0.66 (0.53, 0.8)
	Acetylsalicylic acid	INR≥5	731	0.49 (0.38, 0.63)	0.47 (0.36, 0.6)	0.47 (0.36, 0.6)
	Azathioprine	INR≤1.5	19	0.18 (0.03, 0.64)	0.18 (0.04, 0.55)	0.17 (0.04, 0.53)
	Tramadol	INR≥5	486	0.65 (0.48, 0.86)	0.63 (0.47, 0.82)	0.63 (0.47, 0.82)
**Anti-infectives**						
	Cloxacillin	INR≤1.5	15	0.35 (0.08, 1.2)	0.28 (0.08, 0.84)	0.28 (0.08, 0.85)
	Metronidazole	INR≥5	98	0.58 (0.29, 1.08)	0.47 (0.24, 0.84)	0.47 (0.24, 0.84)
	Rifampicin	INR≤1.5	41	0.36 (0.16, 0.73)	0.28 (0.13, 0.54)	0.28 (0.13, 0.55)
	Teicoplanin	INR≤1.5	48	0.36 (0.18, 0.7)	0.37 (0.19, 0.7)	0.37 (0.19, 0.68)
**Cardiovascular drugs**						
	Amiodarone	INR≥5	856	0.83 (0.67, 1.02)	0.77 (0.62, 0.95)	0.77 (0.62, 0.95)
	Atorvastatin	INR≥5	345	0.66 (0.47, 0.91)	0.64 (0.46, 0.87)	0.64 (0.46, 0.87)
	Bosentan	INR≤1.5	6	0 (0, 0.82)	0^d^	0^d^
	Candesartan	INR≤1.5	225	0.42 (0.31, 0.56)	0.45 (0.33, 0.6)	0.44 (0.33, 0.59)
	Furosemide	INR≤1.5	1955	0.33 (0.29, 0.38)	0.34 (0.3, 0.4)	0.35 (0.3, 0.39)
	Heparin (unfractionated)	INR≥5	294	0.48 (0.32, 0.71)	0.4 (0.27, 0.59)	0.4 (0.27, 0.59)
	Rosuvastatin	INR≥5	181	0.48 (0.28, 0.79)	0.47 (0.28, 0.75)	0.47 (0.28, 0.75)
	Simvastatin	INR≥5	254	0.45 (0.28, 0.68)	0.52 (0.33, 0.79)	0.52 (0.33, 0.79)
**Other drugs**						
	Allopurinol	INR≥5	292	0.64 (0.44, 0.91)	0.6 (0.42, 0.84)	0.6 (0.42, 0.84)
	Omeprazole	INR≥5	155	0.62 (0.36, 1)	0.55 (0.33, 0.86)	0.55 (0.33, 0.86)

^a^OR: odds ratio.

^b^CL: confidence limit.

^c^INR: international normalized ratio.

^d^The 95% CLs were not computable.

**Table 2:** Drugs interacting with VKAs upon discontinuation and that had at least one significant OR (in all cases, 4047 stays are analyzed).

Drug		Outcome	n	OR^a^ (95% CLs^b^)	Adjusted OR (95% CLs)	Stepwise OR (95% CLs)
**Analgesics, anti-inflammatories, and immunologic agents**						
	Acetaminophen	INR^c^≤1.5	251	0.18 (0.12, 0.25)	0.16 (0.11, 0.22)	0.16 (0.11, 0.22)
	Acetylsalicylic acid	INR≤1.5	114	0.2 (0.11, 0.33)	0.21 (0.12, 0.33)	0.2 (0.12, 0.33)
	Dextropropoxyphene	INR≤1.5	22	0.22 (0.05, 0.66)	0.21 (0.06, 0.57)	0.21 (0.06, 0.57)
	Methylprednisolone	INR≤1.5	129	0.19 (0.11, 0.3)	0.19 (0.11, 0.29)	0.18 (0.11, 0.29)
	Tramadol	INR≤1.5	109	0.16 (0.08, 0.27)	0.14 (0.08, 0.24)	0.15 (0.08, 0.24)
**Anti-infectives**						
	Amoxicillin	INR≤1.5	216	0.21 (0.14, 0.3)	0.19 (0.13, 0.27)	0.18 (0.13, 0.26)
	Amoxicillin;clavulanate	INR≤1.5	199	0.25 (0.17, 0.36)	0.23 (0.16, 0.32)	0.22 (0.15, 0.32)
	Ciprofloxacin	INR≤1.5	30	0.11 (0.02, 0.35)	0.09 (0.02, 0.27)	0.09 (0.02, 0.27)
	Clarithromycin	INR≤1.5	11	0.1 (0, 0.69)	0.07 (0, 0.36)	0.06 (0, 0.34)
	Fluconazole	INR≤1.5	16	0.33 (0.08, 1.08)	0.23 (0.06, 0.68)	0.24 (0.07, 0.69)
	Levofloxacin	INR≤1.5	18	0.19 (0.04, 0.69)	0.16 (0.04, 0.5)	0.16 (0.04, 0.49)
	Metronidazole	INR≤1.5	26	0.29 (0.1, 0.76)	0.24 (0.09, 0.57)	0.24 (0.09, 0.58)
	Ofloxacin	INR≤1.5	47	0.3 (0.14, 0.6)	0.27 (0.13, 0.52)	0.27 (0.13, 0.51)
**Cardiovascular drugs**						
	Amiodarone	INR≤1.5	83	0.25 (0.14, 0.44)	0.28 (0.16, 0.47)	0.27 (0.15, 0.46)
	Atorvastatin	INR≤1.5	7	0.16 (0, 1.34)	0.14 (0.01, 0.84)	0.14 (0.01, 0.82)
	Diltiazem	INR≤1.5	20	0.11 (0.01, 0.45)	0.12 (0.02, 0.41)	0.11 (0.02, 0.4)
	Furosemide	INR≥5	246	0.42 (0.25, 0.66)	0.33 (0.2, 0.51)	0.33 (0.2, 0.51)
	Heparin (unfractionated)	INR≤1.5	115	0.19 (0.11, 0.31)	0.18 (0.11, 0.29)	0.18 (0.1, 0.29)
**Other drugs**						
	Allopurinol	INR≤1.5	9	0.28 (0.03, 1.46)	0.23 (0.03, 0.98)	0.24 (0.04, 1.01)
	Ketoconazole	INR≤1.5	7	0 (0, 0.67)	0^d^	0^d^
	Omeprazole	INR≤1.5	26	0.18 (0.04, 0.52)	0.18 (0.05, 0.47)	0.17 (0.05, 0.46)

^a^OR: odds ratio.

^b^CL: confidence limit.

^c^INR: international normalized ratio.

^d^The 95% CLs were not computable.

## Discussion

### Principal Findings

In this study, all the drugs that reportedly interact with VKAs either lacked a statistically significant association or were associated with a statistically significant reduction in risk. Our results suggest that an empirical evaluation of DDIs (as has been suggested for an SPC-CDSS) could help to refine the alerts issued by a CDSS [34]. Our objective was to determine which drugs were associated with an increased risk of bleeding or thrombosis (compared with baseline), rather than to discover which drugs indeed interact with VKAs. It should also be borne in mind that the risk baseline was not zero but corresponded to the actual risk to which inpatients in a given hospital were exposed. This risk was already quite high, and the purpose of a CDSS is to warn physicians when this risk will be accentuated. Hence, our present findings do not contradict the current body of academic knowledge about these drugs.

In all included hospitals, various CDSSs were active before the time of the study. In all of them, the physicians asked for all the alerts to be deactivated. Indeed, physicians were under alert fatigue. Those bad experiences led them to set up the PSIP European Project [9], whose purpose was to find “intelligent” ways to prevent adverse drug events. This paper stands in continuation of the PSIP Project.

### Discussion of the Method

Our study had several strengths. First, the drugs for evaluation were identified through a systematic review of the literature. Second, the study was population-based; in contrast to clinical trials, it was possible to analyze real-life drug administrations, ill-advised drug combinations, and patients with several comorbidities. Along with the INR values, we also took account of the chronology of the drug prescriptions and discontinuations.

Our observational study also had several limitations. First, the number of exposed patients was too small for many drugs. Consequently, our study was not powerful enough to provide firm evidence of an increase or a decrease in the probability of outcomes. This limitation highlights the shortcomings of the SPC-CDSS concept. A reasonable attitude would be to ignore statistical filtering when the number of cases in the learning database is too small. Second, the dose levels of the drugs involved in the DDIs have not been evaluated. Therefore, it cannot be excluded that patients were overdosed or underdosed, which could falsely affect our results. Third, polypharmacy was common (especially in the elderly population; the mean age was 75.9 years) but could not be fully taken into account. Therefore, an outcome counted for one DDI rule could potentially be due to another DDI rule being administered concomitantly to the patient. Fourth, we considered that data were not missing at random and so imputed missing data with normal values; in routine clinical care, nonmeasured parameters are more likely to be normal. Lastly, we used the same onset time (1 day) and discontinuation time (4 days) for each drug, even though the pharmacokinetics differed. Naturally, pharmacokinetics of other possibly interacting drugs are not similar: some of them have a short half-life, and others have a long half-life. Moreover, the kinetics of the interaction cannot be directly inferred from the half-life. Taking this into account would require having a precise description of the mechanisms of all interactions, which is not possible.

The INR is a surrogate marker and does not necessarily reflect clinical outcomes. Indeed, a high INR does not always result in bleeding, nor does a low INR in thrombosis. Furthermore, some DDI interactions for VKAs may lead to clinical outcomes without any change in the INR. However, these clinical outcomes would not have been measured as frequently as the INR was, and the measurements would have been less reliable. Although this would be an issue in automated ADE detection, this approximation is still acceptable when the objective is to filter alerts and identify risk factors.

The number of different patients was 3101 for 4047 stays. Correlation between patients was not taken into account. This attitude can be justified as follows. The calibration of the CDSS is carried out based on statistical individuals that correspond to solicitations of the inference engine and not to physical persons. If some specific patients are more often hospitalized, it makes sense to overweight their statistical properties in the CDSS.

### Discussion of the Results

The statistically significant associations observed for some drugs should not be interpreted as proof of a causal relationship. Indeed, many drugs are associated with specific clinical contexts (ie, indication bias). Those contexts are variously related to the patient (eg, treatments for Alzheimer disease and age), the context of care (eg, antibiotics and bacterial infection), or the prescriber (eg, a cardiologist who is used to prescribing VKAs and avoids DDIs). It should be noted that our present results do not cast doubt on our current body of pharmacological knowledge per se; however, they do challenge the commonly accepted idea whereby this knowledge alone should be used to filter or rank DDI rules [20,21,27]. We suggest that “real-life” empirical probabilities might be more appropriate for these purposes: an alert should be flagged up because there is an actual ADE risk (considering the context, ie, confounding factors, the patient, and the prescriber) and not only a theoretical risk. Perhaps the root of the problem is not so much the DDIs, but the pathological context of the patient. Our hypothesis is that for patients who are doing well, DDIs have a relatively limited impact, due to physiological adaptability. On the other hand, for patients with multiple comorbidities, DDIs have a stronger impact [43,44]. However, using empirical probabilities to automatically filter or rank DDI rules raises a number of issues; the probabilities would have to be updated frequently and computed separately in various contexts [35].

### Potential Impact on Future CDSSs

These probabilities could be used to improve CDSSs in two ways, both of which have been suggested and tested in the literature [35,36,45]: first, to deactivate DDI rules that are associated with an empirical probability below a chosen threshold, and second, to show physicians past cases with outcome to improve their adherence to remaining alerts. The SPC-CDSS concept was recently introduced [34]. The idea is to automatically reuse actual clinical data and search for outcomes (INR≥5, for instance). To prevent the occurrence of an outcome, the SPC-CDSS automatically estimates the conditional probability of an outcome for each rule, assuming that its conditions are met. When the probability is too low (and if there are enough patients), the corresponding alerts are automatically deactivated. In our present work, we used a type 1 error of 5%. A higher threshold (eg, 10%) would remove fewer alerts. The threshold could then be tuned according to the individual physician’s level of risk aversion and alert tolerance. This calculation could also be performed separately for each medical specialty, to take account of the context. This could include latent variables (eg, mean patient characteristics, comorbidities, and the reason for admission), organizational characteristics, and physician characteristics.

As reported in the literature [36,46,47], our present findings confirmed that the reuse of EHR data is an effective way of identifying likely ADEs. Indeed, active postmarket surveillance of drugs must be based on the reuse of data from EHRs and, more specifically, on the inpatient setting; the latter has not been extensively studied [48].

### Conclusion

After calculating the probability that specific medications would interact with VKAs in real life, we found that many of the medications did not show the predicted DDIs. We suggest that EHR data can be automatically mined to filter DDI rules and thus improve CDSSs.

## Appendix

Multimedia Appendix 1 DDI rules (*ATC codes at the time of the data).

## Abbreviations

ADE: adverse drug event
ASAT/ALAT: aspartate transaminase/alanine transaminase
ATC: anatomical therapeutic chemical
CDSS: clinical decision support system
CL: confidence limit
CNS: central nervous system
DDI: drug-drug interaction
EHR: electronic health record
INR: international normalized ratio
Nt-proBNP: N-terminal-pro brain natriuretic peptide
OR: odds ratio
PSIP: patient safety through intelligent procedures
SPC-CDSS: statistically prioritized and contextualized clinical decision support system
TSH: thyroid stimulating hormone
VKA: vitamin K antagonist
