# Supplementary material for: Towards The Automated, Empirical Filtering of Drug-Drug Interaction Alerts in Clinical Decision Support Systems: Historical Cohort Study of Vitamin K Antagonists
Source: JMIR Med Inform. 2021 Jan 20;9(1):e20862. doi: 10.2196/20862 (PMC7857948; doi:10.2196/20862)
Supplement: Multimedia Appendix 1 [file medinform_v9i1e20862_app1.docx]

## Supplementary material:

Appendix 1: DDI rules (*ATC codes at the time of the data)
- Three literature reviews => 148 DDIs (2 initial drugs were mapped to only 1 ATC code, and then 1 DDI rule and 2 drugs can either lower or increase the INR values, and became 4 DDI rules).
- Example of DDI rule #1: it can be read as: [rule #1A] “acetaminophen administration & VKA → INR ≥ 5” and [rule #1B] “acetaminophen discontinuation & VKA → INR ≤ 1.5”

| **N^r^** | **Drug name** | **Outcome if administration** | **Outcome if**  **discontinuation** | **ATC Codes** |
| --- | --- | --- | --- | --- |
| **Analgesics, Anti-inflammatories, Immunologics** | | | | |
| 1 | Acetaminophen | INR ≥ 5 | INR ≤ 1.5 | N02BE01; N02BE51; N02BE71 |
| 2 | Acetylsalicylic acid | INR ≥ 5 | INR ≤ 1.5 | A01AD05; B01AC06; N02BA01; M01BA03; B01AC56; N02BA51; N02BA71; C10BX02; C10BX05; C10BX01; C10BX04 |
| 3 | Azathioprine | INR ≤ 1.5 | INR ≥ 5 | L04AX01 |
| 4 | Celecoxib | INR ≥ 5 | INR ≤ 1.5 | L01XX33; M01AH01 |
| 5 | Cyclosporine | INR ≤ 1.5 | INR ≥ 5 | L04AD01 |
| 6 | Dextropropoxyphene | INR ≥ 5 | INR ≤ 1.5 | N02AC04; N02AC54; N02AC74 |
| 7 | Etodolac | INR ≤ 1.5 | INR ≥ 5 | M01AB08 |
| 8 | Interferon | INR ≥ 5 | INR ≤ 1.5 | L03AB01; L03AB02; L03AB03; L03AB04; L03AB05; L03AB06; L03AB07; L03AB08; L03AB09; L03AB10; L03AB11; L03AB12; L03AB13; L03AB60; L03AB61 |
| 9 | Leflunomide | INR ≥ 5 | INR ≤ 1.5 | L04AA13 |
| 10 | Mercaptopurine | INR ≤ 1.5 | INR ≥ 5 | L01BB02 |
| 11 | Mesalazine (5-ASA) | INR ≤ 1.5 | INR ≥ 5 | A07EC02 |
| 12 | Methylprednisolone | INR ≥ 5 | INR ≤ 1.5 | H02AB04; H02BX01 |
| 13 | Nabumetone | INR ≥ 5 | INR ≤ 1.5 | M01AX01 |
| 14 | Phenylbutazone | INR ≥ 5 | INR ≤ 1.5 | M01AA01; M01BA01 |
| 15 | Piroxicam | INR ≥ 5 | INR ≤ 1.5 | M01AC01 |
| 16 | Rofecoxib | INR ≥ 5 | INR ≤ 1.5 | M01AH02 |
| 17 | Sulfasalazine | INR ≤ 1.5 | INR ≥ 5 | A07EC01 |
| 18 | Sulindac | INR ≥ 5 | INR ≤ 1.5 | M01AB02 |
| 19 | Tolmetin | INR ≥ 5 | INR ≤ 1.5 | M01AB03 |
| 20 | Tramadol | INR ≥ 5 | INR ≤ 1.5 | N02AX02; N02AX52 |
| 21 | Trastuzumab | INR ≥ 5 | INR ≤ 1.5 | L01XC03; L01XC14 |
| **Anti-infectives** | | | | |
| 22 | Amoxicillin | INR ≥ 5 | INR ≤ 1.5 | J01CA04; J01CR02; A02BD01; A02BD03; A02BD04; A02BD05; A02BD06; A02BD07; A02BD10 |
| 23 | Amoxicillin + β-lactamase inhibitor | INR ≥ 5 | INR ≤ 1.5 | J01CR02 |
| 24 | Azithromycin | INR ≥ 5 | INR ≤ 1.5 | J01FA10 |
| 25 | Cefamandole | INR ≥ 5 | INR ≤ 1.5 | J01DC03 |
| 26 | Cefazolin | INR ≥ 5 | INR ≤ 1.5 | J01DB04 |
| 27 | Chloramphenicol | INR ≥ 5 | INR ≤ 1.5 | J01BA01 |
| 28 | Ciprofloxacin | INR ≥ 5 | INR ≤ 1.5 | J01MA02 |
| 29 | Clarithromycin | INR ≥ 5 | INR ≤ 1.5 | J01FA09; A02BD06; A02BD07; A02BD05; A02BD04 |
| 30 | Cloxacillin | INR ≤ 1.5 | INR ≥ 5 | J01CF02 |
| 31 | Dicloxacillin | INR ≤ 1.5 | INR ≥ 5 | J01CF01 |
| 32 | Doxycycline | INR ≥ 5 | INR ≤ 1.5 | A01AB22; J01AA02 |
| 33 | Efavirenz | INR ≥ 5 | INR ≤ 1.5 | J05AG03 |
| 34 | Erythromycin | INR ≥ 5 | INR ≤ 1.5 | J01FA01 |
| 35 | Etravirine | INR ≥ 5 | INR ≤ 1.5 | J05AG04 |
| 36 | Fluconazole | INR ≥ 5 | INR ≤ 1.5 | J01RA07; J02AC01 |
| 37 | Fosamprenavir | INR ≤ 1.5 | INR ≥ 5 | J05AE07 |
| 38 | Gatifloxacin | INR ≥ 5 | INR ≤ 1.5 | J01MA16 |
| 39 | Griseofulvin | INR ≤ 1.5 | INR ≥ 5 | D01BA01 |
| 40 | Isoniazid | INR ≥ 5 | INR ≤ 1.5 | J04AC01; J04AC51; J04AM01; J04AM02; J04AM03; J04AM04; J04AM05; J04AM06; J04AM01; J04AM04 |
| 41 | Itraconazole | INR ≥ 5 | INR ≤ 1.5 | J02AC02 |
| 42 | Levamisole | INR ≥ 5 | INR ≤ 1.5 | P02CE01 |
| 43 | Levofloxacin | INR ≥ 5 | INR ≤ 1.5 | J01MA12; J01RA05; A02BD10 |
| 44 | Metronidazole | INR ≥ 5 | INR ≤ 1.5 | A02BD08; A02BD03; A02BD02; J01XD01; P01AB01; P01AB01; A02BD01 |
| 45 | Miconazole, oral gel | INR ≥ 5 | INR ≤ 1.5 | A01AB09 |
| 46 | Miconazole, topical gel | INR ≥ 5 | INR ≤ 1.5 | D01AC02; D01AC52 |
| 47 | Miconazole, vaginal suppositories | INR ≥ 5 | INR ≤ 1.5 | G01AF04 |
| 48 | Moxifloxacin | INR ≥ 5 | INR ≤ 1.5 | J01MA14; S01AE07 |
| 49 | Nafcillin | INR ≤ 1.5 | INR ≥ 5 | J01CF06 |
| 50 | Nalidixic acid | INR ≥ 5 | INR ≤ 1.5 | J01MB02 |
| 51 | Nevirapine | INR ≥ 5 | INR ≤ 1.5 | J05AG01 |
| 52 | Nevirapine | INR ≤ 1.5 | INR ≥ 5 | J05AG01 |
| 53 | Norfloxacin | INR ≥ 5 | INR ≤ 1.5 | J01MA06 |
| 54 | Ofloxacin | INR ≥ 5 | INR ≤ 1.5 | J01MA01 |
| 55 | Ribavirin | INR ≤ 1.5 | INR ≥ 5 | J05AB04 |
| 56 | Rifampicin | INR ≤ 1.5 | INR ≥ 5 | J04AB02; J04AM02; J04AM05; J04AM06 |
| 57 | Ritonavir | INR ≥ 5 | INR ≤ 1.5 | J05AR10; J05AE03 |
| 58 | Ritonavir | INR ≤ 1.5 | INR ≥ 5 | J05AR10; J05AE03 |
| 59 | Saquinavir | INR ≥ 5 | INR ≤ 1.5 | J05AE01 |
| 60 | Sulfisoxazole | INR ≥ 5 | INR ≤ 1.5 | J01EB05 |
| 61 | Teicoplanin | INR ≤ 1.5 | INR ≥ 5 | J01XA02 |
| 62 | Terbinafine | INR ≥ 5 | INR ≤ 1.5 | D01BA02 |
| 63 | Terbinafine | INR ≤ 1.5 | INR ≥ 5 | D01BA02 |
| 64 | Tetracycline | INR ≥ 5 | INR ≤ 1.5 | J01AA01; J01AA02; J01AA03; J01AA04; J01AA05; J01AA06; J01AA07; J01AA08; J01AA09; J01AA10; J01AA11; J01AA12; J01AA20; J01AA56; J01RA08; A02BD02; A02BD08 |
| 65 | Tranexamic acid | INR ≥ 5 | INR ≤ 1.5 | B02AA02 |
| 66 | Trimethoprim; Sulfamethoxazole | INR ≥ 5 | INR ≤ 1.5 | J01EE01 |
| 67 | Voriconazole | INR ≥ 5 | INR ≤ 1.5 | J02AC03 |
| **Cardiovascular Drugs** | | | | |
| 68 | Amiodarone | INR ≥ 5 | INR ≤ 1.5 | C01BD01 |
| 69 | Atorvastatin | INR ≥ 5 | INR ≤ 1.5 | C10AA05 |
| 70 | Bezafibrate | INR ≥ 5 | INR ≤ 1.5 | C10AB02 |
| 71 | Bosentan | INR ≤ 1.5 | INR ≥ 5 | C02KX01 |
| 72 | Candesartan | INR ≤ 1.5 | INR ≥ 5 | C09CA06; C09DB07; C09DA06 |
| 73 | Chelation therapy | INR ≤ 1.5 | INR ≥ 5 | V03AB09; V03AB03; M01CC01; V03AC01; V03AC02; V03AC03 |
| 74 | Cholestyramine | INR ≤ 1.5 | INR ≥ 5 | C10AC01 |
| 75 | Clofibrate | INR ≥ 5 | INR ≤ 1.5 | C10AB01; C10AB03 |
| 76 | Diltiazem | INR ≥ 5 | INR ≤ 1.5 | C08DB01 |
| 77 | Disopyramide | INR ≥ 5 | INR ≤ 1.5 | C01BA03 |
| 78 | Dronedarone | INR ≥ 5 | INR ≤ 1.5 | C01BD07 |
| 79 | Ezetimibe | INR ≥ 5 | INR ≤ 1.5 | C10AX09 |
| 80 | Fenofibrate | INR ≥ 5 | INR ≤ 1.5 | C10AB11; C10AB05; C10BA03; C10BA04 |
| 81 | Fluvastatin | INR ≥ 5 | INR ≤ 1.5 | C10AA04 |
| 82 | Furosemide | INR ≤ 1.5 | INR ≥ 5 | C03CA01; C03CB01; C03EB01 |
| 83 | Gemfibrozil | INR ≥ 5 | INR ≤ 1.5 | C10AB04 |
| 84 | Heparin (unfractionated) | INR ≥ 5 | INR ≤ 1.5 | B01AB01; B01AB51 |
| 85 | Indometacin | INR ≥ 5 | INR ≤ 1.5 | C01EB03; M01AB01 |
| 86 | Lovastatin | INR ≥ 5 | INR ≤ 1.5 | C10AA02; C10BA01 |
| 87 | Metolazone | INR ≥ 5 | INR ≤ 1.5 | C03BA08; C03EA12 |
| 88 | Orlistat | INR ≥ 5 | INR ≤ 1.5 | A08AB01 |
| 89 | Propafenone | INR ≥ 5 | INR ≤ 1.5 | C01BC03 |
| 90 | Propranolol | INR ≥ 5 | INR ≤ 1.5 | C07AA05; C07FA05; C07BA05 |
| 91 | Quinidine | INR ≥ 5 | INR ≤ 1.5 | C01BA01; C01BA51; C01BA71 |
| 92 | Rosuvastatin | INR ≥ 5 | INR ≤ 1.5 | C10AA07 |
| 93 | Simvastatin | INR ≥ 5 | INR ≤ 1.5 | C10AA01; C10BX01; C10BA02; C10BA04; C10BX04; A10BH51 |
| 94 | Telmisartan | INR ≤ 1.5 | INR ≥ 5 | C09CA07; C09DB04; C09DA07 |
| 95 | Ticlopidine | INR ≥ 5 | INR ≤ 1.5 | B01AC05 |
| 96 | Ubidecarenone | INR ≤ 1.5 | INR ≥ 5 | C01EB09 |
| **CNS Drugs** | | | | |
| 97 | Barbiturates | INR ≤ 1.5 | INR ≥ 5 | N01AF01; N01AF02; N01AF03; N01AG01; N03AA01; N03AA02; N03AA03; N03AA04; N03AA30; N05CA01; N05CA02; N05CA03; N05CA04; N05CA05; N05CA06; N05CA07; N05CA08; N05CA09; N05CA10; N05CA11; N05CA12; N05CA15; N05CA16; N05CA19; N05CA20; N05CA21; N05CA22; N05CB01; N05CB02 |
| 98 | Carbamazepine | INR ≤ 1.5 | INR ≥ 5 | N03AF01 |
| 99 | Chlordiazepoxide | INR ≤ 1.5 | INR ≥ 5 | N05BA02 |
| 100 | Choral hydrate | INR ≥ 5 | INR ≤ 1.5 | N05CC01; N05CC03 |
| 101 | Citalopram | INR ≥ 5 | INR ≤ 1.5 | N06AB04 |
| 102 | Disulfiram | INR ≥ 5 | INR ≤ 1.5 | N07BB01 |
| 103 | Duloxetine | INR ≥ 5 | INR ≤ 1.5 | N06AX21 |
| 104 | Entacapone | INR ≥ 5 | INR ≤ 1.5 | N04BX02 |
| 105 | Felbamate | INR ≥ 5 | INR ≤ 1.5 | N03AX10 |
| 106 | Fluoxetine | INR ≥ 5 | INR ≤ 1.5 | N06AB03; N06CA03 |
| 107 | Fluvoxamine | INR ≥ 5 | INR ≤ 1.5 | N06AB08 |
| 108 | Methylphenidate | INR ≥ 5 | INR ≤ 1.5 | N06BA04 |
| 109 | Phenytoin | INR ≥ 5 | INR ≤ 1.5 | N03AB02; N03AB52 |
| 110 | Propofol | INR ≤ 1.5 | INR ≥ 5 | N01AX10 |
| 111 | Quetiapine | INR ≥ 5 | INR ≤ 1.5 | N05AH04 |
| 112 | Ropinirole | INR ≥ 5 | INR ≤ 1.5 | N04BC04 |
| 113 | Sertraline | INR ≥ 5 | INR ≤ 1.5 | N06AB06 |
| 114 | Trazodone | INR ≤ 1.5 | INR ≥ 5 | N06AX05 |
| **Other Drugs** | | | | |
| 115 | Acarbose | INR ≥ 5 | INR ≤ 1.5 | A10BF01; A10BD17 |
| 116 | Allopurinol | INR ≥ 5 | INR ≤ 1.5 | M04AA01 |
| 117 | Anabolic steroids | INR ≥ 5 | INR ≤ 1.5 | A14AA01; A14AA02; A14AA03; A14AA04; A14AA05; A14AA06; A14AA07; A14AA08; A14AA09; A14AB01; A14AB02; A14AB03 |
| 118 | Cimetidine | INR ≥ 5 | INR ≤ 1.5 | A02BA01; A02BA51 |
| 119 | Danazol | INR ≥ 5 | INR ≤ 1.5 | G03XA01 |
| 120 | Ethanol | INR ≥ 5 | INR ≤ 1.5 | D08AX08; V03AB16; V03AZ01; R02AA03; C04AC02; C10AD05 |
| 121 | Etretinate | INR ≤ 1.5 | INR ≥ 5 | D05BB01 |
| 122 | Fluorouracil | INR ≥ 5 | INR ≤ 1.5 | L01BC02; L01BC52 |
| 123 | Gemcitabine | INR ≥ 5 | INR ≤ 1.5 | L01BC05 |
| 124 | Glucagon | INR ≥ 5 | INR ≤ 1.5 | H04AA01 |
| 125 | Ifosphamide | INR ≥ 5 | INR ≤ 1.5 | L01AA06 |
| 126 | Influenzae vaccine | INR ≤ 1.5 | INR ≥ 5 | J07BB01; J07BB02; J07BB03 |
| 127 | Ketoconazole | INR ≥ 5 | INR ≤ 1.5 | D01AC08; G01AF11; J02AB02 |
| 128 | Levonorgestrel | INR ≥ 5 | INR ≤ 1.5 | G03AC03; G03AD01; G03FA11; G03FB09; G03AA07; G03AB03 |
| 129 | Omeprazole | INR ≥ 5 | INR ≤ 1.5 | A02BC01; A02BD01; A02BD05 |
| 130 | Oxolamine | INR ≥ 5 | INR ≤ 1.5 | R05DB07 |
| 131 | Paclitaxel | INR ≥ 5 | INR ≤ 1.5 | L01CD01; L01CD03 |
| 132 | Raloxifene | INR ≤ 1.5 | INR ≥ 5 | G03XC01 |
| 133 | Sucralfate | INR ≤ 1.5 | INR ≥ 5 | A02BX02 |
| 134 | Sulfamethoxazole | INR ≥ 5 | INR ≤ 1.5 | J01EC01 |
| 135 | Sulfinpyrazone | INR ≥ 5 | INR ≤ 1.5 | M04AB02 |
| 136 | Sulfinpyrazone | INR ≤ 1.5 | INR ≥ 5 | M04AB02 |
| 137 | Tamoxifen | INR ≥ 5 | INR ≤ 1.5 | L02BA01 |
| 138 | Tolterodine | INR ≥ 5 | INR ≤ 1.5 | G04BD07 |
| 139 | Topical salicylates (anti-acne) | INR ≥ 5 | INR ≤ 1.5 | D02AF; D01AE12 |
| 140 | Troglitazone | INR ≥ 5 | INR ≤ 1.5 | A10BG01 |
| 141 | Zafirlukast | INR ≥ 5 | INR ≤ 1.5 | R03DC01 |
| **No ATC code*** | | | | |
| 142 | Propoxyphene | INR ≥ 5 | INR ≤ 1.5 | *NA* |
| 143 | CMF | INR ≥ 5 | INR ≤ 1.5 | *NA* |
| 144 | Lopinavir; ritonavir | INR ≤ 1.5 | INR ≥ 5 | *NA* |
| 145 | Zileuton | INR ≥ 5 | INR ≤ 1.5 | *NA* |
| 146 | Doxifluridine | INR ≥ 5 | INR ≤ 1.5 | *NA* |
| 147 | Etoposide ; carboplatine | INR ≥ 5 | INR ≤ 1.5 | *NA* |
| 148 | Methimazole | INR ≤ 1.5 | INR ≥ 5 | *NA* |
